# Comments on “The mRNA and Protein Levels of the Glycolytic Enzymes Lactate Dehydrogenase A (LDHA) and Phosphofructokinase Platelet (PFKP) Are Good Predictors of Survival Time, Recurrence, and Risk of Death in Cervical Cancer Patients”

**DOI:** 10.1002/cam4.70691

**Published:** 2025-02-20

**Authors:** Hadi Raeisi Shahraki

**Affiliations:** ^1^ Department of Epidemiology and Biostatistics, Faculty of Health Shahrekord University of Medical Sciences Shahrekord Iran

Dear Editor,

I meticulously read the paper by Bolaños‐Suárez et al. that was recently published online in *Cancer Medicine*. In this article, the authors tried to cluster patients with cervical cancer using the information of 14 glycolytic genes expression [1]. Undoubtedly, their study makes a valuable contribution to the area and using multivariate statistical analysis is interesting, but some methodological issues need to be taken into account.

*The Subjective Determination of the Number of Clusters*:

The optimal number of cluster determinations is a fundamental issue in cluster analysis. Choosing any desired number of clusters by researchers is subjective and leads to bias by ignoring the nature of the data structure.

To address this issue, using direct methods such as the silhouette method or incorporating statistical tests such as gap statistics was suggested. The *NbClust* package in R software provides more than 30 indices for determining the optimal number of clusters [2].

*Wrong Specification of Three Clusters Based on the Displayed Dendrogram*:

The tree‐based representation of hierarchical cluster analysis called dendrogram was displayed in a figure by Bolaños‐Suárez et al. To identify three clusters, we must cut the dendrogram at a certain height which corresponds to just three main branches of the tree (the red line in Figure 1). As illustrated in Figure 1, the obtained clusters are very different from the proposed clusters by Bolaños‐Suárez et al.

**FIGURE 1:**
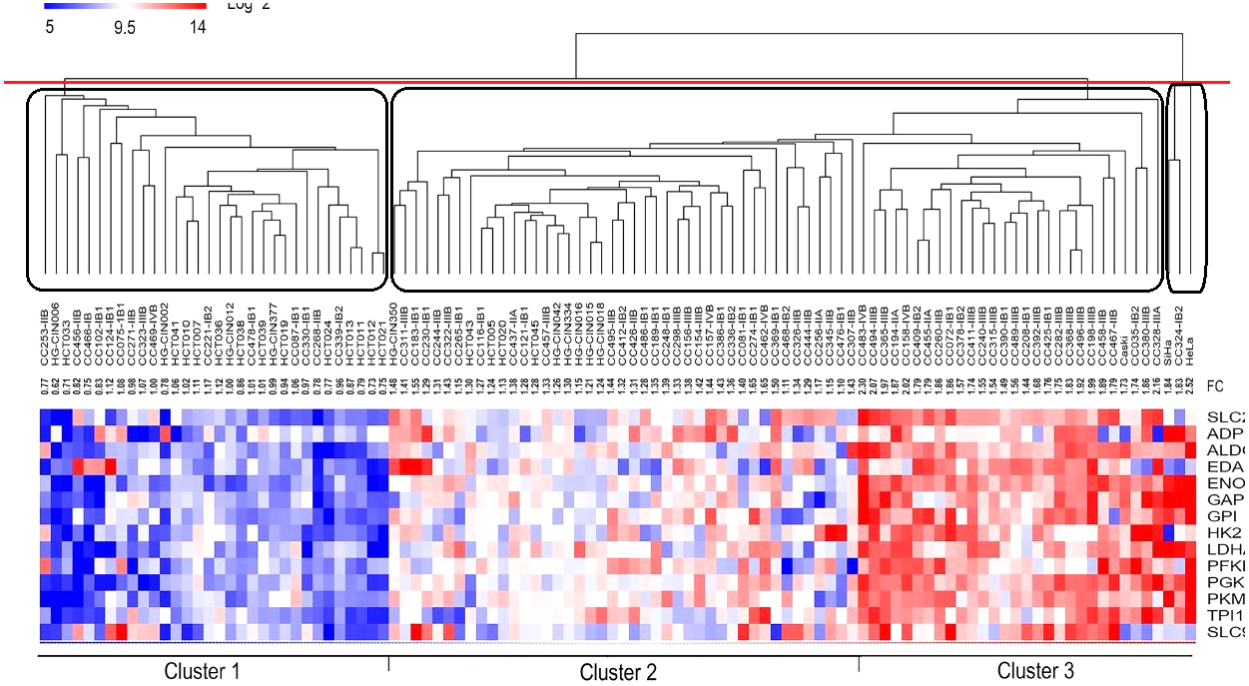
The true assignment versus the proposed assignment of observations into three clusters.

In conclusion, checking the obtained results for consistency or biological relevance is necessary. Moreover, researchers must be aware that determining the optimal number of clusters is not a subjective issue, and using appropriate statistical indices is highly suggested.

I would like to thank again Bolaños‐Suárez et al. for their valuable article and for sharing with us their in‐depth investigation and analysis.

## Author Contributions

**Hadi Raeisi Shahraki:** conceptualization (lead), data curation (lead), formal analysis (equal), funding acquisition (equal), investigation (equal), methodology (equal), project administration (equal), resources (equal), software (equal), supervision (equal), validation (equal), visualization (equal), writing – original draft (equal), writing – review and editing (equal).

## Conflicts of Interest

The author declares no conflicts of interest.

## Data Availability

The author has nothing to report.
